# Role of Signaling Molecules in Mitochondrial Stress Response

**DOI:** 10.3389/fgene.2018.00225

**Published:** 2018-07-10

**Authors:** Shauna Hill, Kavithalakshmi Sataranatarajan, Holly Van Remmen

**Affiliations:** ^1^Aging and Metabolism Research Program, Oklahoma Medical Research Foundation, Oklahoma City, OK, United States; ^2^Department of Cell Systems & Anatomy, University of Texas Health at San Antonio, San Antonio, TX, United States; ^3^Department of Pathology, University of Washington, Seattle, WA, United States; ^4^Oklahoma City VA Medical Center, Oklahoma City, OK, United States

**Keywords:** mitochondria, stress response, longevity, signaling peptides, retrograde response

## Abstract

Mitochondria are established essential regulators of cellular function and metabolism. Mitochondria regulate redox homeostasis, maintain energy (ATP) production through oxidative phosphorylation, buffer calcium levels, and control cell death through apoptosis. In addition to these critical cell functions, recent evidence supports a signaling role for mitochondria. For example, studies over the past few years have established that peptides released from the mitochondria mediate stress responses such as the mitochondrial unfolded protein response (UPR^MT^) through signaling to the nucleus. Mitochondrial damage or danger associated molecular patterns (DAMPs) provide a link between mitochondria, inflammation and inflammatory disease processes. Additionally, a new class of peptides generated by the mitochondria affords protection against age-related diseases in mammals. In this short review, we highlight the role of mitochondrial signaling and regulation of cellular activities through the mitochondrial UPR^MT^ that signals to the nucleus to affect homeostatic responses, DAMPs, and mitochondrial derived peptides.

## Introduction

**FIGURE 1:**
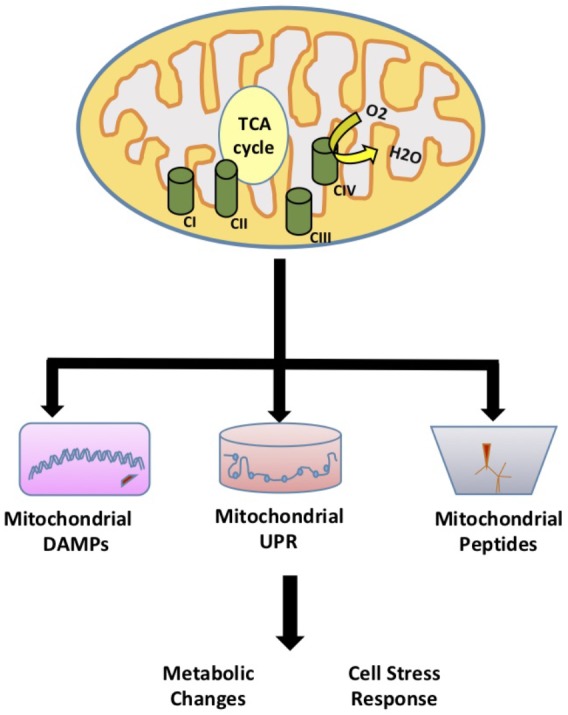
Mitochondrial Signaling. The role of mitochondria in signaling and regulation of cellular activities through the mitochondrial unfolded protein response (UPR^MT^) that signals to the nucleus to affect homeostatic responses, damage or danger associated molecular patterns (DAMPs), and mitochondrial derived peptides (MDPs).

Mitochondria are essential double membrane cellular organelles that provide the energy to the cell through oxidative phosphorylation. Mitochondria also play a key role in cellular function and homeostasis through adenosine triphosphate (ATP) production, calcium homeostasis, apoptosis signaling, and fatty acid oxidation. In addition, mitochondria modulate redox signaling through the production of reactive oxygen species (ROS). While the role of mitochondrial ROS signaling in modulation of cellular processes has been established, recently several studies have highlighted additional signaling roles for mitochondria that are not initiated by ROS. The idea that mitochondria can regulate cellular metabolism through mitochondrial derived signaling was first described in yeast. Using mitochondrial DNA depleted *rho°* yeast cells, Ron Butow discovered yeast mitochondria have adapted a mitochondria-to-nucleus signal transduction pathway termed the retrograde response to induce the transcription of nuclear-encoded mitochondrial genes and alleviate mitochondrial stress (Parikh et al., 1987). Higher organisms have adapted a similar retrograde signaling response known as the mitochondrial unfolded protein response (UPR^MT^). This response is initiated by the accumulation of unfolded proteins in the mitochondria resulting in the induction of UPR^MT^ components (Benedetti et al., 2006). In addition, mitochondria can regulate cell function and metabolism by signaling pathways that involve mitochondrial-derived peptides and other mitochondrial signals such as mitochondrial damage-associated molecular patterns (mito-DAMPs). In this review, we highlight the role of mitochondrial signaling and regulation of cellular activities through the mitochondrial UPR^MT^ that signals to the nucleus to affect homeostatic responses, damage or danger associated molecular patterns (DAMPs), and mitochondrial derived peptides (MDPs) (**Figure 1**).

### Mitochondrial Unfolded Protein Response (UPR^MT^)

The UPR^MT^ signaling pathway was first elucidated in *C. elegans.* Mitochondrial stress generated from misfolded proteins was shown to activate the adenosine triphosphate (ATP)-dependent mitochondrial protease, ClpP, to cleave misfolded proteins (Benedetti et al., 2006; Haynes et al., 2007). The exported peptides in turn activate the nuclear translocation of ATFS-1 (Activating Transcription Factor associated with Stress) where it subsequently activates UBL-5 to form a complex with transcription factor DVE-1 to transcriptionally activate *UPR^MT^* genes, heat shock protein 6 (*hsp-6*) and *hsp-10* (Martinus et al., 1996; Haynes et al., 2007, 2010). Subsequent studies found that under normal conditions ATFS-1 accumulates in the mitochondria, but during mitochondrial stress (ETC dysfunction, ROS production, proteotoxic stress, etc.) ATFS-1 accumulates in the cytosol and is transported to the nucleus to induce the expression of mitochondrial stress proteins (Nargund et al., 2012). A recent study in *C. elegans* showed that the H3K27 demethylases jmjd-1.2 and jmjd-3.1 are required for the UPR^MT^ suggesting that the UPR^MT^ can also be regulated at the epigenetic level (Merkwirth et al., 2016). In another recent report in *C. elegans*, the UPR^MT^ was proposed to promote the propagation of deleterious mtDNA rather than eliminating deleterious mtDNA (Lin et al., 2016). These findings suggest that the UPR^MT^ could be used as a therapeutic target for mtDNA disorders and associated diseases (further reviewed in Tian et al., 2016).

The regulation of the UPR^MT^ in mammals is not fully understood, but a few key components have been identified in mammals with the first report by Zhao et al. (2002) showing that UPR^MT^ components are elevated in mitochondrial DNA (mtDNA) depleted mammalian cells. The mammalian transcription factors, CHOP and C/EBPβ were implicated as putative transcription factors for this pathway based on conserved regulatory element in promoters of the UPR^MT^ related genes (Aldridge et al., 2007). In this model, mitochondrial proteotoxic stress mediates CHOP transcriptional activation through the Jnk/c-Jun pathway (Weiss et al., 2003; Jaeschke et al., 2006). This pathway is conserved in mammals as the Jnk/c-Jun pathway is required for transcriptional activation of CHOP and UPR^MT^ induction in mitochondrial ornithine transcarbamylase (OTC) mutant Cos-7 cells under proteotoxic stress (Rath et al., 2012). A recent study by Fiorese et al. (2016) identified the mammalian transcription factor ATF-5 to be the functional ortholog of the *C. elegans* ATFS-1 transcription factor. The transcription factor, ATF-4 has also been recently shown to be an important mediator of mitochondrial stress response (Quiros et al., 2017). However, ATF-4 was not shown to regulate UPR^MT^ genes but cytoprotective genes that regulate cellular metabolism. Studies in mouse tissue show a consistent correlation between H3K27 demethylases and UPR^MT^ transcripts supporting the notion that UPR^MT^ gene regulation is through demethylases proceeding the retrograde signaling from the mitochondria (Merkwirth et al., 2016).

Studies from our laboratory have shown that mice harboring a null mutation in the *SURF1* gene, an electron transport chain complex IV assembly factor, show an induction of the UPR^MT^ in several tissues that is associated with a number of beneficial phenotypes including increased insulin sensitivity, mitochondrial biogenesis, and increased resistance to oxidative stress in cultured fibroblasts (Deepa et al., 2013; Lin et al., 2013; Pulliam et al., 2014; Pharaoh et al., 2016). Shpilka and Haynes (2018) have extensively reviewed the implications of the UPR^MT^ in aging and age-related diseases. These studies suggest a potential link between the UPR^MT^, metabolism and stress resistance in mammals.

#### Signaling Peptides Associated With the Mitochondrial Unfolded Protein Response

The mitochondrial UPR^MT^ is of interest as a direct form of communication between the mitochondria and the nucleus. However, a study by Durieux et al. (2011) suggested that mitochondrial stress could be signaled through cell non-autonomous mechanisms. Specifically, studies in *C. elegans* showed that mitochondrial proteotoxic stress restricted to neuronal tissue induced the mitochondrial UPR^MT^ in a heterologous tissue, the intestine (Durieux et al., 2011). These findings suggest that the UPR^MT^ can be activated in a cell non-autonomous fashion. Interestingly, components of the UPR^MT^, ATFS-1, and DVE-1 in the neurons are required to induce the UPR^MT^ in the intestine. Further studies have been conducted to determine the signaling factor released by the neurons to induce the UPR^MT^ in the intestine. One study suggested that distal activation of the UPR^MT^ requires UNC-31 mediated secretion of serotonin (Berendzen et al., 2016). However, this study used a general proteotoxic stress model, PolyQ40, and not a mitochondrial specific model. In another study, neuropeptides were investigated to determine if they mediate the UPR^MT^ in a cell non-autonomous fashion using a neuronal specific CRISPR-Cas9 approach to manipulate mitochondrial stress (Shao et al., 2016). Neuropeptides are released from dense core vesicles derived at the synapse and may function as hormones to systemically alter cellular activities. This study showed that deletion of 6 out of the 103 reported neuropeptides blocked the neuronal-specific mitochondrial stress induction of the UPR^MT^ in the intestine. These neuropeptides include INS-17, INS-34, FLP-2, FLP-15, NLP-10, and NLP-28. Subsequently, only constitutive overexpression of *flp-2* was shown to induce the UPR^MT^ on its own. These studies suggest that FLP-2 is released by neurons during mitochondrial stress to signal for the induction of the UPR^MT^ in the periphery. Interestingly, overexpression of *flp-2* activates the UPR^MT^ but does not extend lifespan. This supports other studies where the UPR^MT^ was shown to not have a casual effect on lifespan (Bennett et al., 2014). The identification of these signaling molecules could have significant implications in the development of therapies to target mitochondrial disease and improve healthspan.

### Mitochondrial Damage Associated Molecular Patterns

**FIGURE 2:**
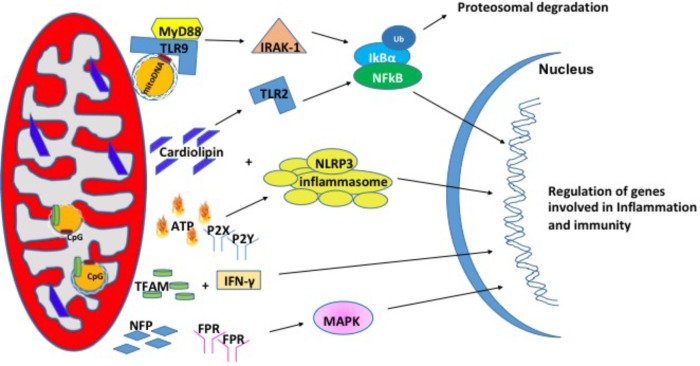
Mitochondrial damage-associated molecular patterns (Mito-DAMPs). Mitochondrial damage associated molecular patterns are in the form of mitochondrial DNA (mtDNA), cardiolipin, ATP, TFAM, and NFP. In response to stress, mitochondria release these small molecules to signal for the nuclear transcription of genes involved in inflammation and immunity.

In recent years, studies have shown that mitochondria can modulate inflammation and the immune response through the release of signaling factors known as Mito-DAMPs (Zhang et al., 2010). Under physiological conditions, damage associated molecular patterns (DAMPs) are not recognized by immune system. During cellular stress or tissue injury, these molecules can be released from dying cells or damaged extracellular matrix in to the extracellular environment (Wenceslau et al., 2014; Land, 2015). During pathological insults, Mito-DAMPs have consequences on the innate immune response and inflammation (Krysko et al., 2011). Mito-DAMPs include mtDNA, TFAM, cardiolipin, ATP, and *N*-formyl peptides (NFPs) (Nakahira et al., 2015) (**Figure 2**).

#### Mitochondrial DNA (mtDNA)

Mitochondrial DNA encodes essential protein subunits of the oxidative phosphorylation system that play primary role in respiration and ATP production. Oxidized or fragmented mtDNA is released from damaged mitochondria and evokes an immune response (Garcia and Chavez, 2007; Nakahira et al., 2011). The unmethylated CpG site of the mtDNA binds to TLR9 and activates a downstream cascade of reactions by recruiting the adaptor proteins MyD88; IL-1R-associated kinase (IRAK4), interferon regulatory factor-7 (IRF7), tumor-necrosis factor-alpha receptor activated factor-6 (TRAF6) (Hacker et al., 2000; Huang and Yang, 2010). These events are followed by activation of ERK, p38, and IkB pathways that finally culminate in the transcription of inflammatory genes (Yi and Krieg, 1998; Deguine and Barton, 2014). mtDNA levels in the plasma of the elderly (age 90 years) correlated with pro-inflammatory cytokines like TNF-α, IL-1β, IL-6, IL-1Rα, and RANTES (Pinti et al., 2014) in glomerular kidney disease (Bao et al., 2016), in heart (Bliksoen et al., 2016), and in sporadic ALS (Vielhaber et al., 2000). In addition, circulating mtDNA levels are increased in acute liver injury (Marques et al., 2012), hypertension (McCarthy et al., 2015), acute kidney injury (Tsuji et al., 2016). Apart from the increased levels in the circulation, mtDNA copy number has also been shown to be increased in aging skeletal muscle (Masser et al., 2016), lung tissue (Lee et al., 2000), in cancer (Cormio et al., 2012) and the level of mtDNA in the synovial fluid was shown to correlate with the severity of rheumatoid arthritis (Hajizadeh et al., 2003). A recent study from Sandler et al. (2018) suggests that mtDNA may be an early biomarker in post-operative complications such as cardiopulmonary bypass surgery (Sandler et al., 2018) and in traumatic brain injury (Walko et al., 2014). Thus, mtDNA is associated with a number of diseases and may also play a role in pathologies of aging.

#### TFAM

Mitochondrial transcription factor, TFAM is an abundant protein that plays an important role in regulating mtDNA content. TFAM is tightly bound to mtDNA and amplifies the mtDNA induced TLR9 immune response (Julian et al., 2012). TFAM can act directly as a DAMP. For example, treatment of THP1 monocytic cells with TFAM resulted in increased expression IL-1β, IL-6, and IL-8 (Little et al., 2014). In another study, serum TFAM levels were doubled after hemorrhagic shock and resuscitation in Sprague-Dawley rats (Chaung et al., 2012), which in turn induces inflammatory responses. The impact of TFAM in neurodegeneration diseases has been reviewed briefly in Kang et al. (2018).

#### Cardiolipin

Cardiolipin is a phospholipid present in the inner mitochondrial membrane that is a central player in various processes like mitochondrial calcium uniporter, mitochondrial protein kinase C signaling, mitophagy, cytochrome c release during apoptosis and inflammasome activation (Dudek, 2017). Upon translocation to the outer membrane, cardiolipin activates NLRP3 promoting inflammation (Iyer et al., 2013). During cell injury, cardiolipin undergoes oxidation, and released into the extracellular environment where it acts as a mito-DAMPs (Claypool and Koehler, 2012). In support of this, patients with pneumonia show high concentration of cardiolipin in lung fluid (Ray et al., 2010) and patients with mitochondrial disease showed higher cardiolipin content in the skeletal muscle (Schlame et al., 1999). There are several reports elucidating the mechanism by which cardiolipin acts as mito-DAMP under different pathological conditions. In a study with autoimmune disease patients, cardiolipin signals through TLR2-PI3K-PKN1-AKT-p38MAPK-NFkB pathway to activate antigen presenting cells (Cho et al., 2018). In another study involving pneumonia, cardiolipin inhibits interleukin (IL)-10 production by inducing the SUMOylation of the nuclear receptor PPAR gamma. PPAR gamma SUMOylation results in binding of the repressive complex NCOR/HDAC3 to IL-10 promotor but not the TNF promoter thereby efficiently inhibiting IL-10 production (Chakraborty et al., 2017).

#### Adenosine Triphosphate (ATP)

Adenosine triphosphate is the currency of intracellular energy. ATP plays an important role in glycolysis, TCA cycle, and beta oxidation. Apart from being used as source of energy, it plays a crucial role in biochemical signaling pathways including DNA and RNA synthesis and protein synthesis. Under physiological conditions, there remains a balance between ATP secretion and its extracellular concentration. When this balance is lost, extracellular ATP (eATP) plays a toxic role. eATP is upregulated in diabetic nephropathy (Chen et al., 2013), hypertension (Ji et al., 2012), induces vascular inflammation, atherosclerosis (Stachon et al., 2016), and lung inflammation (Riteau et al., 2010). eATP can be fueled by stimuli such as membrane damage, mechanical stress, excitation of neural tissue (Fields, 2011). eATP binds to the purinergic receptor subtype P2X or P2Y receptor (Surprenant and North, 2009; Erlinge, 2011) and can play a key role in regulation of vascular endothelium, pain and inflammatory responses. Ectonucleotidases CD39 and CD73 can degrade eATP to ADP, AMP and adenosine and each of these molecules can bind to P2 receptors and activate responses related to tissue damage and inflammation (Wilkin et al., 2001; Bours et al., 2006). eATP has been shown to activate the NLRP3 inflammasome that results in the release of IL-18 (Amores-Iniesta et al., 2017) through P2X7 in allograft rejection (Amores-Iniesta et al., 2017). Importantly, P2X7 -receptor inhibitors CE224, AZD9056, and GSK1482160 are available in clinical use as immunomodulatory agents (Vergani et al., 2014).

#### *N*-Formyl Peptides (NFPs)

*N*-Formyl peptides are a class of peptides that are produced by bacterial cells and mitochondria suggesting their involvement in host defense against bacterial infection and the clearance of damaged cells. NFPs have a high affinity binding site for FPR, NFP (Boulay et al., 1990; Rabiet et al., 2005). FPRs are a class of transmembrane G protein-coupled receptors that have three isoforms (FPR1, FPR2, and FPR3) and are highly expressed on monocytes and neutrophils suggesting their involvement in immune cell response (Waller et al., 2004). NFPs have been shown to drive neutrophil activation through MAPK and ERK1/2 signaling pathways (Hazeldine et al., 2015). In cell culture models, the addition of mito-DAMPs initiated chemotaxis, production of TNFα as well as a rapid release of ROS (Rabiet et al., 2005; Friedenberg et al., 2016). The induction of chemotaxis via NFPs is dependent on calcium influx through FPRs (Le et al., 2002).

A number of studies have shown that NFPs are elevated in response to post-traumatic injury and diseased models (Zhang et al., 2010; Dorward et al., 2017). These elevated levels lead to systemic inflammatory response syndrome (SIRS) (Hazeldine et al., 2015). For example, elevated NFPs lead to airway contraction and lung inflammation (Wenceslau et al., 2016). On the other hand, the loss of NFP binding receptor, FPR increases the susceptibility to *L. monocytogenes* in mice (Liu et al., 2012). Inflammation is positively correlated with aging and this relationship is coined ‘inflamm-aging’ (Franceschi et al., 2000). Therefore, NFP signaling may be a therapeutic target to reduce systemic inflammation and increase healthspan in humans.

### Mitochondrial Derived Peptides (MDPs)

Mitochondrial derived peptides are a novel class of mitochondrial signaling peptides that are encoded by short open reading frames (sORFs) in the mitochondrial genome. MDPs regulate a wide range of cellular signaling pathways and have an implicated role in aging. In this section, we review known MDPs and their role in cellular activities.

#### Humanin

Humanin was the first discovered MDP using a functional expression screen for peptides that could suppress neuronal cell death induced by Aβ (Hashimoto et al., 2001). Humanin is a 24-amino acid peptide that’s encoded by the mitochondrial 16S rRNA. Humanin has gained significant recognition for its cytoprotective cellular activities and protection against a wide range of pathologies. A number of studies implicate humanin as a potential therapeutic target for a range of diseases. For example, synthetic humanin improves memory deficits and reduced Aβ plaques in rodent models of Alzheimer’s disease (Niikura et al., 2011; Zhang et al., 2012; Chai et al., 2014). Studies have shown that humanin protects against stroke in mice (Xu et al., 2006), ameliorates atherosclerotic plaque formation (Oh et al., 2011), improves insulin sensitivity in rodent models of diabetes (Hoang et al., 2010), and affords cardioprotection against myocardial ischemia (Thummasorn et al., 2016). Additionally, humanin restores cellular ATP levels in cells isolated from human patients affected by the mitochondrial disease, mitochondrial encephalomyopathy with lactic acidosis and stroke-like episodes (Kariya et al., 2005). The cytoprotective activities of humanin provide this protection in a number of disease models.

Humanin elicits cytoprotection inter- and intracellular via anti-apoptotic effects and binding to two plasma membrane receptors. Humanin interacts via binding with IGFBP-3, Bax, and tBid (Guo et al., 2003; Ikonen et al., 2003; Zhai et al., 2005). In cell culture studies, humanin shows BAX dependent anti-apoptotic effects against serum deprivation and tumor necrosis factor (Guo et al., 2003; Zhai et al., 2005). Humanin also exhibits anti-apoptotic effects by binding to IGFBP-3 to block nuclear translocation required to induce apoptosis (Ikonen et al., 2003). More recently, humanin is shown to prevent endoplasmic reticulum (ER)-stress induced apoptosis by mediating ER-mitochondrial cross talk for cell survival (Matsunaga et al., 2016; Sreekumar et al., 2017). As a result, humanin suppresses apoptosis and promotes cell survival during oxidative and ER stress. Humanin also protects from apoptosis through the preservation of mitochondrial homeostasis in responses to cellular stress (Klein et al., 2013; Thummasorn et al., 2016; Cui et al., 2017). In addition, circulating humanin binds to two plasma membrane receptors to initiate a number of cytoprotective activities. One of the two receptors is the trimeric CNTFR/gp130/WSX-1 receptor (Hashimoto et al., 2001, 2009; Ying et al., 2004). Humanin induces the hetero-oligomerization of CNTFR, gp130, and WSX-1 and subsequently binds to the CNTFR/gp130/WSX-1 receptor to activate the JAK-STAT pathway (Kim et al., 2016). Humanin-induced neuroprotection requires the activation of Stat3 via the CNTFR/gp130/WSX-1 receptor (Hashimoto et al., 2005). Activation of Stat3 by humanin also improves diabetes in a mouse model by inhibiting pancreatic β-cell apoptosis and improving glucose tolerance (Hoang et al., 2010). Humanin protects against damage from Aβ42 by binding to the formyl peptide receptor-like 1/2 (FRPL1/2) receptor (Ying et al., 2004). It’s hypothesized that humanin exerts its neuroprotection by competing with Aβ42 for the binding to FRPL1/2 since Aβ42 accumulation is linked to FRPL1/2 binding.

Due to the universal cytoprotective activities of humanin, it has been proposed to have therapeutic potential to enhance human healthspan. In fact, humanin has been linked to aging. Humanin levels significantly decline with age in humans and rodent models (Muzumdar et al., 2009; Bachar et al., 2010; Hoang et al., 2010). Offspring of centenarians have been shown to have higher levels of humanin compared to the rest of the aging population (Muzumdar et al., 2009). Together, these studies suggest that retaining humanin levels with age may promote healthy aging.

#### MOTS-c (Mitochondrial Open Reading Frame of the 12S rRNA-c)

MOTS-c is a 16-amino-acid peptide encoded by 12S rRNA sORF in the mtDNA that has been proposed to play a role in regulation of metabolism (Lee et al., 2015). The MOTS-c transcript is polyadenylated and subsequently exported to the cytoplasm for translation. Phylogenetic studies show that MOTS-c is highly conserved across species (Lee et al., 2015). In mice, MOTS-c is present in a number of high-energy demanding tissues with the main targets being skeletal muscle and adipose tissue. MOTS-c is also present in the circulation of humans and mice (Lee et al., 2015).

Microarray analysis in two different mammalian cell culture lines demonstrated that MOTS-c regulates global gene expression specifically affecting metabolic and inflammatory related genes (Lee et al., 2015). The alterations in global metabolic gene expression translate to changes in the metabolite profile. Metabolomic studies showed that MOTS-c reduces metabolites involved in purine and dipeptide metabolism and increases metabolites involved in acylcarnitine and methionine metabolism. MOTS-c also promotes the biosynthesis of the AMPK activator, AICAR (Lee et al., 2015). These alterations in the metabolomic profile induced by MOTS-c suggest a potential target for metabolic disease as well as aging.

Studies have shown that MOTS-c can protect against a number of pathologies. MOTS-c treated mice have reduced body weight, food intake, and blood glucose. MOTS-c promotes insulin sensitivity and protects from high-fat diet induced insulin resistance and obesity in mice (Lee et al., 2015). Specifically, MOTS-c promotes insulin sensitivity in the skeletal muscle by stimulating glucose clearance as measured with the hyperinsulinemic-euglycemic clamp technique (Lee et al., 2015). MOTS-c also has a protective role in ovariectomy-induced bone loss. MOTS-c treatment inhibited receptor activator of nuclear factor-κB ligand (RANK) induced osteoclast differentiation (Ming et al., 2016). The protective effects of MOTS-c on bone loss are partially dependent on MOTS-C mediated AMPK activation since an AMPK inhibitor partially reversed these effects (Ming et al., 2016). Additionally, MOTS-c administration reduced basal circulating levels of IL-6 and TNFα (Lee et al., 2015).

Analyses of the mitochondrial genome from an exceptionally long-lived Japanese population suggest a role for MOTS-c in human longevity (Fuku et al., 2015a). Mitochondrial genome analysis from a Northeast Asian population identified a m.1382A > C polymorphism located in the MOTS-c encoding mtDNA (Fuku et al., 2015a,b). This single nucleotide polymorphism results in a single amino acid substitution predicted to have function consequences on the small peptide. This alteration in MOTS-C could contribute to the exceptional longevity observed in the Northeast Asian population. Together, these data suggest that MOTS-c may be a potential therapeutic target to improve healthspan.

#### Small Humanin-Like Peptides (SHLPs)

An *in silico* prediction analyses of sORFs identified six novel peptides named small humanin-like peptides (SHLPs) 1–6 (Cobb et al., 2016). The existence of these novel SHLPs were validated by mRNA and peptide expression levels in cells, tissues, and plasma. The origin of SHLPs 1–6 has been determined by RT-PCR. SHLPs 1, 4, 5, and 6 were confirmed to be mitochondrial in origin. However, SHLPs 2 and 3 were amplified from both mitochondrial and nuclear cDNA, which leaves the possibility that SHLPs 2 and 3 are not exclusively mitochondrial in origin.

Cell viability and apoptosis studies revealed that SHLPs 2 and 3 promotes cell viability and protection against cellular apoptosis in NIT-1 and 22Rv1, mouse beta and human prostate cancer cells, respectively (Cobb et al., 2016). One method in which SHLPs 2 and 3 promote cell viability is through the reduction of ROS production. The peptides, SHLPs 2 and 3 increase basal oxygen consumption and ATP production (Cobb et al., 2016). Furthermore, SHLPs 2 and 3 promotes adipocyte differentiation of 3T3L pre-adipocytes. SHLPs 2 and 3 activate ERK and STAT-3 signaling (Cobb et al., 2016).

Similar to humanin and MOTS-c, SHLP 2 circulating levels decline with age making it a potential therapeutic target for age-related diseases (Cobb et al., 2016). In fact, SHLP 2 has similar effects on insulin sensitivity as humanin and MOTS-c. In Sprague-Dawley rats, SHLP 2 enhanced the exogenous glucose infusion rate by 50% and promoted glucose uptake by peripheral tissues measured with the hyperinsulinemic-euglycemic clamp technique (Cobb et al., 2016). These findings suggest that SHLP 2 is an insulin sensitizer and implicates a therapeutic potential for SHLP 2 for diabetes. Additionally, SHLP 2 supplementation prevented Aβ-induced neuronal cell death (Cobb et al., 2016). SHLP 2 may exert neuroprotection similar to humanin via the activation of STAT-3. Future studies should investigate the effects of SHLP 2 in Alzheimer’s disease and diabetic mammalian models. Furthermore, it is important to establish the role of SHLP 2 in modulating healthspan as well as characterize the biological and physiological effects of the other SHLPs.

## Summary

Since the initial finding of mitochondrial nuclear signaling in yeast, our understanding of the potential for mitochondrial to act in a cell signaling capacity has expanded significantly. A similar mitochondrial-nuclear signaling pathway, the UPR^MT^, was demonstrated in *C. elegans* and more recently studies have demonstrated that mitochondrial stress restricted to neuronal tissue induced the UPR^MT^ in a heterologous tissue, the intestine. These findings suggest that there is not only intracellular signaling resulting in UPR^MT^ activation, but that there is also signaling between/among tissues resulting in UPR^MT^ activation. This is a novel concept that has important implications for extending our understanding of the role of mitochondria in cellular function and resistance to stress. Mitochondrial peptides have also been shown to regulate cytoprotective activities and mitochondrial stress responses in mammals. For example, a recently discovered MDP, humanin, is released during mitochondrial dysfunction resulting in an increase of cytoprotective activities and providing protection against a range of pathologies. Since the discovery of humanin, MOTS-C and a class of six other mitochondrial derived peptides (SHLPs) have been identified. Similar to humanin, these peptides have shown to afford cytoprotection but not much is known. Future work will likely continue to demonstrate exciting new signaling and regulatory functions for the mitochondria.

## Author Contributions

SH, KS, and HVR wrote the text. KS prepared the figures.

## Conflict of Interest Statement

The authors declare that the research was conducted in the absence of any commercial or financial relationships that could be construed as a potential conflict of interest.
